# Renal and Inflammation Markers—Renalase, Cystatin C, and NGAL Levels in Asymptomatic and Symptomatic SARS-CoV-2 Infection in a One-Month Follow-Up Study

**DOI:** 10.3390/diagnostics12010108

**Published:** 2022-01-04

**Authors:** Natalia Serwin, Elżbieta Cecerska-Heryć, Ewa Pius-Sadowska, Karol Serwin, Anna Niedźwiedź, Magda Wiśniewska, Marta Roszak, Bartłomiej Grygorcewicz, Edyta Skwirczyńska, Bogusław Machaliński, Barbara Dołęgowska

**Affiliations:** 1 Department of Laboratory Medicine, Pomeranian Medical University in Szczecin, al. Powstańców Wlkp. 72, 70-111 Szczecin, Poland; elzbieta.cecerska.heryc@pum.edu.pl (E.C.-H.); martaroszak95@gmail.com (M.R.); bartlomiej.grygorcewicz@pum.edu.pl (B.G.); barbara.dolegowska@pum.edu.pl (B.D.); 2 Department of General Pathology, Pomeranian Medical University in Szczecin, al. Powstańców Wlkp. 72, 70-111 Szczecin, Poland; ewapius@wp.pl (E.P.-S.); boguslaw.machalinski@pum.edu.pl (B.M.); 3 Department of Infectious, Tropical Diseases and Immune Deficiency, Pomeranian Medical University in Szczecin, ul. Arkońska 4, 71-455 Szczecin, Poland; karol.serwin@pum.edu.pl; 4 Independent Public Regional Hospital in Szczecin, ul. Arkońska 4, 71-455 Szczecin, Poland; ania.niedzwiedz@gmail.com; 5 Department of Nephrology, Transplantology, and Internal Medicine, Pomeranian Medical University in Szczecin, al. Powstańców Wlkp. 72, 70-111 Szczecin, Poland; mwisniewska35@gmail.com; 6 Department of History of Medicine and Medical Ethics, Pomeranian Medical University in Szczecin, ul. Rybacka 1, 70-205 Szczecin, Poland; edyta.skwirczynska@pum.edu.pl

**Keywords:** renalase, NGAL, cystatin C, COVID-19, SARS-CoV-2

## Abstract

The aim of our study was to evaluate the influence of asymptomatic infection and the occurrence of symptomatic COVID-19 on specific biochemical, renal, and immune parameters—renalase, neutrophil gelatinase-associated lipocalin (NGAL) cystatin C (CysC), and creatinine—and their weekly fluctuations during a one-month observation period in COVID-19 patients admitted to hospital. The study involved 86 individuals: 30 patients with diagnosed COVID-19, 28 people with asymptomatic infection confirmed with IgG antibodies—the IG(+) group—and 28 individuals without any (IgG, IgE) anti-SARS-CoV-2 antibodies—the IG(−) group. In the COVID-19 group, blood was drawn four times: (1) on day 0/1 after admission to hospital (C1 group), (2) 7 days later (C7 group), (3) 14 days later (C14 group), and (4) 28 days later (C28 group). In the IG(−) and IG(+) groups, blood was drawn once. There were no significant differences in creatinine, Cys C, and uric acid between any of the analyzed groups. NGAL levels were significantly higher in IG(+) and at all time-points in the COVID-19 groups than in controls. A similar observation was made for renalase at the C7, C14, and C28 time-points. Plasma renalase, NGAL, and CysC are unrelated to kidney function in non-critically ill COVID-19 patients and those with asymptomatic infection. Renalase and NGAL are most likely related to the activation of the immune system rather than kidney function. Asymptomatic SARS-CoV-2 infection causes a rise in plasma NGAL levels similar to those observed in symptomatic COVID-19 patients. Therefore, more attention should be paid to tracking and monitoring the health of these people.

## 1. Introduction

Severe acute respiratory syndrome coronavirus 2 (SARS-CoV-2) and the resulting COVID-19 are currently one of the most critical epidemiological problems. SARS-CoV-2 is a member of the *Coronaviridae* family, which are large, enveloped, positive-sense, single-strand RNA viruses that are particularly difficult to control by prophylactic vaccines and virus-specific drugs. It can infect multiple cell types, but lung type-II pneumocytes and ciliated epithelial cells are the primary sites of virus replication; later, infected cells undergo apoptotic or necrotic death and attract the innate immune cells and activate them to secrete pro-inflammatory cytokines [1]. The emergence of the virus and COVID-19 led to changes in the established methods of diagnosis and treatment of many diseases as its effects are related to multi-organ changes. Much attention has been focused on the consequences associated with the respiratory and cardiovascular systems. The virus causes severe respiratory complications and has an affinity with the angiotensin-converting enzyme 2 (ACE2) receptor [2]. Moreover, many COVID-19 patients are experiencing an increase in hepatic parameters, probably caused by activation of the immune system, as in other COVID-19-related diseases [3]. COVID-19 has also proven adverse neurological and psychiatric outcomes—a large, often difficult-to-diagnose problem, reaching approximately one third of convalescents [4]. The impact on other tissues and organs is still a subject for analysis, some of which will require many years of observation. Some recent studies have shown that renal function in COVID-19 patients deteriorates, and AKI is the one of the main observed renal complications, immediately after the electrolyte disturbance [5]. This deterioration might be a result of direct action on kidney cells and interaction with the ACE2 receptor, widely expressed in proximal epithelial cells, vascular endothelial, smooth muscle cells, and podocytes, or indirect as a consequence of acute or chronic inflammation, immune response, and inflammatory cells’ infiltration during infection [6]. At the same time, research in this area is still developing, and their results indicate many, often divergent, observations, starting with no significant influence of COVID-19 on renal function and ending with the assumption that new onset of kidney disease may occur [7]. These are primarily based on the basic established methods of determining and describing kidney function. However, they should be expanded over time with new factors and potential biomarkers that aspire to be markers or predictors of damage to these organs.

One such factor is renalase (RNLS), a small flavoprotein produced mainly by the kidney. Some investigations show that RNLS might be an “organolase” as the *RNLS* gene is expressed in many other cells and tissues, including the nervous system, endocrinal and digestive tract organs, lungs, and heart in humans and other mammals, where its function is not entirely understood [8]. RNLS shows both intracellular and extracellular activity. Intracellular RNLS acts as an enzyme that, in the presence of a FAD cofactor, oxidizes 2- and 6-DHNAD(P) to β-NAD(P)H, a biologically active form. This action prevents the toxicity resulting from the inhibition of many β-NAD(P)H-dependent enzymes and reactions. In turn, extracellular renalase and RP-200 and RP-220 peptides, which are fragments of the protein, activate some of the signaling pathways, including Akt and MAP kinases, promoting cell survival. This activity is mediated by the binding of renalase to its recently discovered receptor—plasma membrane Ca2+-ATPase-4b (PMCA4b)—which is the main form of this pump in erythrocytes. These cells are also involved in renalase transport [9]. Despite discrepancies in observed serum renalase levels in humans, most analyses indicate that the RNLS concentration is significantly increased in people with chronic kidney disease (CKD). As for many other markers, this relationship would demonstrate the usefulness of RNLS concentration assessment in the diagnosis and prognosis of kidney diseases and accompanying disorders.

Neutrophil gelatinase-associated lipocalin (NGAL), also known as lipocalin 2 (LCN2), is an important immune element with indicator functions. NGAL is an iron chelator, a small protein transporting small ligands such as vitamins and pheromones. It has three isoforms, 25 kDa, 45 kDa, and 145 kDa, all produced by neutrophils [10]. In the kidney, it is produced in the proximal and distal tubules of the nephron, almost exclusively in the 25 kDa monomeric form [11]. NGAL is a relatively good, sensitive urinary marker of kidney damage in acute kidney injury, post-contrast nephropathy, polycystic kidney disease (ADPKD), or glomerulonephritis [12,13,14,15]. Both urine and plasma NGAL have been used as early biomarkers for AKI in patients undergoing heart surgery, which was always associated with neutrophil activation [10]. However, the NGAL concentration changes significantly each time neutrophils are activated, which might be confounding in interpretation. Due to the repeatedly demonstrated sensitivity of the tests, plasma NGAL in renal patients has been called, by some researchers, an “expensive [alternative to] creatinine” [16].

Contrary to the factors described above, cystatin C (CysC) is a well-known molecule increasingly used to evaluate and describe kidney function. CysC is produced by most nucleated cells and serves as a good indicator of renal function as it is less influenced by the body muscle mass than creatinine levels. It is also more sensitive than serum creatinine to early changes in GFR. In normal conditions, it is easily filtered in the glomeruli and completely reabsorbed by tubular epithelial cells and degraded within cells. In healthy adults, CysC levels should not exceed 1 mg/L [17]; however, the exact physiological concentration ranges of CysC differ in various scientific literature sources.

The impact of SARS-CoV-2 on pulmonary and cardiac systems has attracted remarkable attention. However, the effects of this virus on renal function are still undervalued. They need more investigation, since most SARS-CoV-2 infections are asymptomatic or result in mild symptoms and are not usually subject to any observations or clinical control. Most studies focus on the course of the COVID-19 disease caused by the virus; asymptomatic people are assessed for the possibility of spreading the virus but rarely undergo diagnostic or screening tests for possible complications of the infection. The aim of our study was to evaluate the influence of asymptomatic infection and occurrence of symptomatic COVID-19 on specific biochemical, renal, and immune parameters—renalase, NGAL, cystatin C, and creatinine—and their weekly fluctuations during a one-month observation period in COVID-19 patients admitted to hospital.

## 2. Materials and Methods

### 2.1. Patients

**Figure 1:**
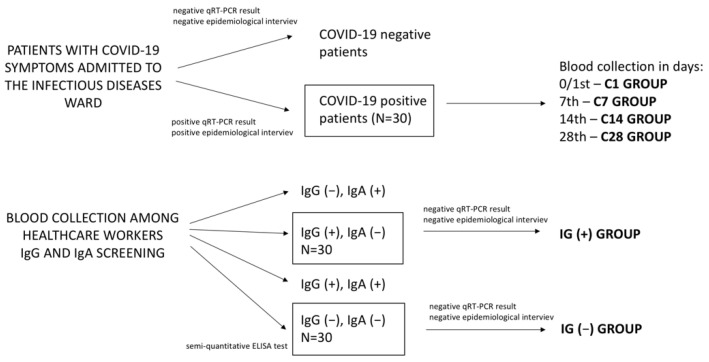
A brief diagram of the selection of patients and material collection.

The study involved 86 individuals: 30 patients (14 women and 16 men) with diagnosed COVID-19, 28 people with asymptomatic infection confirmed with IgG antibodies (19 women and 9 men)—the IG(+) group—and 28 individuals without any (IgG, IgE) (10 women and 18 men) anti-SARS-CoV-2 antibodies—the IG(−) control group. The IG(+) group was selected among healthcare workers of the Independent Public Clinical Hospital No. 2 PUM in Szczecin, particularly exposed to contact with the virus. All material in this group was collected in June 2020. The COVID-19 group was chosen from among patients on the infectious ward who underwent blood sampling four times: (1) on day 0/1, immediately after admission to hospital (C1 group), (2) 7 days later (C7 group), (3) 14 days later (C14 group), and (4) 28 days later (C28 group). The collection of blood from this group was performed systematically from June 2020 to June 2021. All patients left the hospital after 1–2 weeks; hence, blood was collected later by a special departing team at the patient’s home. None of the patients were connected to a ventilator. All patients were able to communicate freely and gave informed consent to participate in the study after answering questions from a survey prepared for the purposes of the study, asked by the interviewer before blood sampling. In the IG(+) group and IG(−) control group, the blood was drawn only once. A brief diagram of the selection of patients and material collection is presented in Figure 1.

Diagnosis of COVID-19 was based on symptoms of the disease, positive quantitative real-time PCR (qRT-PCR) test, and epidemiological history. Individuals from IG(−) and IG(+) groups had a negative qRT-PCR test and negative epidemiological history. The IgG and IgA antibodies specific to SARS-CoV-2 in the IG(+) and IG(−) groups were determined by the ELISA method in the Department of Laboratory Diagnostics of Independent Public Clinical Hospital No.2 PUM in Szczecin. Among people with positive results for antibodies, people with only IgG antibodies, which are antibodies of the so-called “late-stage” of infection, were selected.

Exclusion criteria were: 1. severe renal or hepatic dysfunction that may disturb the production, transport, and elimination of antibodies in the body; 2. acquired immunodeficiency virus (HIV) and congenital humoral and cellular immunodeficiencies; 3. persons who did not give their written consent to participate in the study.

All patients completed the designed questionnaire and gave informed consent. The study was approved by the Bioethical Committee of the Pomeranian Medical University (No. KB-0012/83/2020).

### 2.2. Methods

All immunoenzymatic and biochemical analyses were performed in blood plasma obtained from whole K3EDTA blood by centrifugation (1000× *g*, room temperature, 10 min). Levels of renalase were measured using an EIAab Science (Wuhan, China) ELISA kit specific for renalase. NGAL and cystatin C levels were evaluated using R&D Systems (Minneapolis, MN, USA) ELISA tests. Biochemical tests were performed using the spectrophotometric method and ready-made test kits (BioMaxima, Lublin, Poland).

### 2.3. Statistical Analysis

All variables were expressed as means ± standard deviation and medians (lower quartile–upper quartile). Categorical variables were assessed using the Chi-square test and Fisher’s exact test if appropriate. To compare the difference for each biochemical parameter and biomarker over time in the COVID-19 group, we used repeated-measures ANOVA with Tukey comparisons. To assess differences between unpaired groups, we used U Mann–Whitney test when analyzing two groups, and Kruskal–Wallis ANOVA with post-hoc Dunn’s test for more than two groups. Correlations were evaluated using Spearman’s correlation rank test. *p* values < 0.05 were considered to be statistically significant. Statistical analyses were performed using R statistical software ver. 4.1.0, (R Foundation for Statistical Computing, Vienna, Austria).

## 3. Results

### 3.1. COVID-19 Patient Characteristics

In the COVID-19 patients’ group, four patients had chronic kidney disease before SARS-CoV-2 infection. Two of them experienced worsening of kidney function during the disease. General worsening of renal parameters was observed in ten patients. Two of them developed AKI, diagnosed, according to Kidney Disease: Improving Global Outcomes (KDIGO) recommendations, as an increase in creatinine by 0.3 mg/dL or more within 48 h or a 1.5-fold increase within a week. In six COVID-19 patients, an increase in hepatic parameters was observed. Four patients also had a pulmonary embolism.

Apart from the mentioned disorders and diseases, most of the patients (*n* = 26) before COVID-19 disease had underlying/concomitant diseases: diabetes mellitus type 2 (*n* = 4), arterial hypertension (*n* = 10), bronchial asthma (*n* = 4), obesity (*n* = 4), fatty liver (*n* = 3), hypothyroidism (*n* = 2), gallbladder stones (*n* = 2), depression (*n* = 2), and others, occurring singly or together. They included psoriatic arthritis, age-related macular degeneration (AMD), heart, liver, and pancreas disorders, history of cancer, and others. The worsening of kidney function, AKI, increase in hepatic parameters, and pulmonary embolism were further used to group patients for statistical analysis purposes.

In the IG(+) and IG(−) controls, none of the individuals reported health problems or the presence of concomitant diseases that would exclude them from participation in the study (see exclusion criteria). Two IG(+) and two IG(−) individuals were excluded from the further analysis due to the withdrawal of consent to participate in the study.

### 3.2. Biomarker Measurement

**Figure 2:**
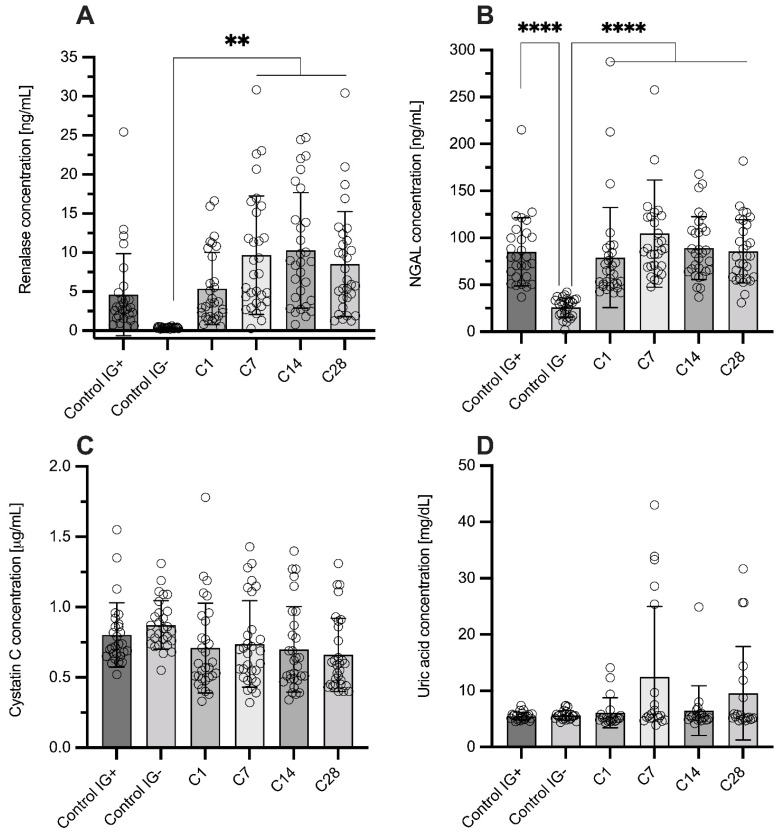
Renalase (**A**), neutrophil gelatinase-associated lipocalin (**B**), cystatin C (**C**), and uric acid (**D**) levels in analyzed groups. ** *p* < 0.01; **** *p* < 0.001.

Levels of measured parameters are shown in Table 1. None of the biochemical parameters—creatinine, uric acid, glucose, albumin, triglycerides, total and HDL cholesterol—differed significantly between any of the analyzed groups (Figure 2 and Figure S1 in Supplementary Material data). NGAL in the control group was significantly lower than in any COVID-19 or IG(+) group. There was no difference in plasma NGAL levels between the IG(+) group and COVID-19 patients at any of the time-points (Figure 2B). The highest concentration was observed on day 7, but it was not a statistically significant relationship. RNLS levels in the control group were negligible and, similarly to NGAL, significantly lower than in any COVID-19 group apart from C1 (Figure 2A). The highest renalase levels were observed on the 14th day after admission to the hospital. There also was no difference between the IG(+) group and any of the COVID-19 groups. As with creatinine and uric acid, cystatin C levels did not differ between groups (IG(+), IG(−), C1-C28) (Figure 2C). Moreover, none of the participants, including COVID-19 patients, had significantly elevated cystatin C levels above normal values (>1.1 mg/L).

When divided based on the occurrence of worsening of kidney function, AKI, an increase in hepatic parameters, or pulmonary embolism, no differences among COVID-19 patients in any of the analyzed parameters were observed.

**Figure 3:**
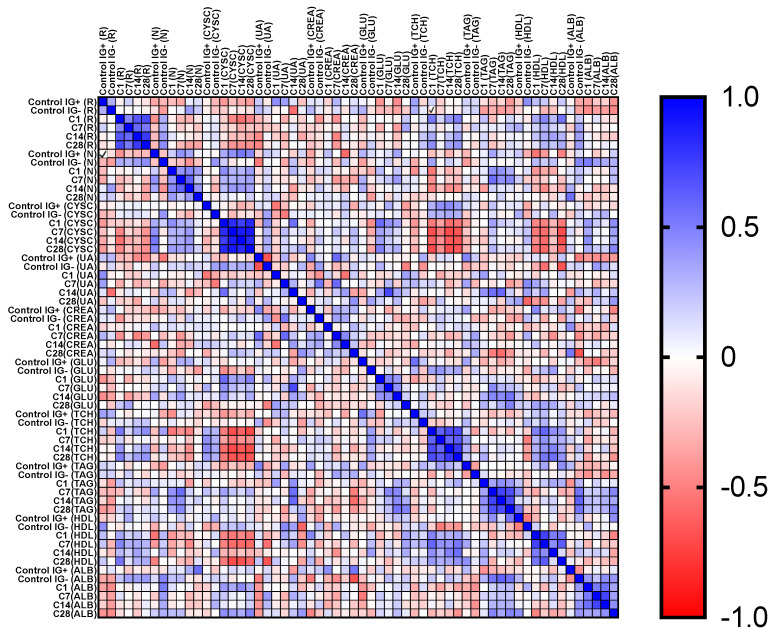
Correlation matrix heatmap for analyzed parameters in all groups; R—renalase; N—neutrophil gelatinase-associated lipocalin (NGAL); CYSC—cystatin C; UA—uric acid; CREA—creatinine; GLU—glucose; TCH—total cholesterol; TAG—triglycerides; HDL—high-density lipoprotein cholesterol; ALB—albumin.

General correlations between analyzed parameters in all groups were presented as a heatmap (Figure 3). In the IG(−) group, renalase correlated significantly with NGAL (Rs = −0.34, *p* < 0.05). In the IG(+) group, such a correlation was not observed but it was close to significance (Rs = −0.39, *p* = 0.07). This relation was lost among COVID-19 patients in each time-point, apart from day 28 (Rs = −0.35, *p* < 0.05). NGAL also correlated with cystatin C in the IG(−) group (Rs = 0.41, *p* < 0.05), and C1 and C14 time-points (Rs = 0.44, *p* < 0.05, and Rs = 0.40, *p* < 0.05, respectively).

**Figure 4:**
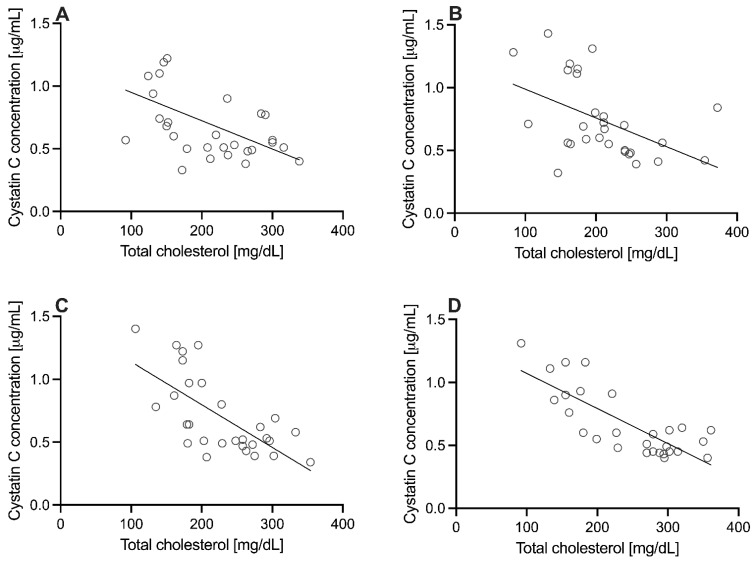
Scatterplots for correlations between cystatin C and total cholesterol in the COVID-19 group. (**A**)—C1 time-point; (**B**)—C7 time-point; (**C**)—C14 time-point; (**D**)—C28 time-point.

An interesting observation was a significant, moderate negative correlation between cystatin C and total cholesterol in each of the time-points in the COVID-19 group (C1: Rs = −0.51, *p* < 0.01; C7: Rs = −0.52, *p* < 0.01; C14: Rs = −0.65, *p* < 0.01; C28: Rs = −0.66, *p* < 0.01). This also translated into the HDL cholesterol fraction, for which the same, significant, negative correlation was observed except for point C14 (C1: Rs = −0.47, *p* < 0.01; C7: Rs = −0.58, *p* < 0.01; C14: Rs = −23, *p* = 0.22; C28: Rs = −0.60, *p* < 0.01). Both mentioned relationships were not observed in the IG(−) and IG(+) groups. Scatterplots for correlations between cystatin C and total cholesterol in the COVID-19 groups are shown in Figure 4.

## 4. Discussion

Deterioration in kidney function is another emerging health problem in COVID-19 patients, and the predictive value of serum cystatin C in the prognosis of these patients is rarely reported. Some studies have indicated that serum cystatin C is a predictor of AKI and death in critically ill patients with acute cerebral infarction, acute myocardial infarction, heart failure, or sepsis [18]. Interestingly, cystatin C values in healthy adults range from 0.6 to 1.0 mg/L, while COVID-19 patients with cystatin C levels of 0.80 mg/L or greater were shown to be at a high risk of death [19]. In this context, it should be noted that some conditions and medications might lower the concentration of cystatin C. In our study, two patients had extremely low levels of cystatin C (below 0.4 mg/L over the whole observation). This was most likely due to multiple comorbidities, including cancer, hypertension, condition after acute myocardial infarction and angioplasty, hepatitis C and others, and their treatment. Therefore, the predictive value of this molecule must always be carefully considered. In our study, assessment of values of plasma cystatin C in evaluating the risk of death among patients who left the hospital in good general condition could not be determined or was specified as “having no effect” on mortality. According to our results, kidney function, assessed based on both creatinine and cystatin C levels, did not deteriorate significantly in the whole group during the one-month observation period. Patients who developed AKI during the study also had cystatin C, uric acid, and creatinine levels that were insignificantly different from those in other patients.

The negative correlation between plasma cystatin C and cholesterol, including the HDL fraction, has not yet been described in the scientific literature, pointing to the basis of such a relationship. A significant proportion of people in the COVID-19 group had elevated cholesterol values above normal (>200 mg/dL) at each time-point. At the same time, mean cystatin C concentrations were much lower than 0.80 mg/L observed in IG(−) and IG(+) groups, defined as the threshold of increased risk of death. Some studies show that, in normal and pathophysiological conditions, cystatin C is more often positively related to triglycerides, eventually HDL [20,21]. Changes in both mentioned parameters are observed in many diseases, especially in diabetes, HIV infection, or cancer; considering the production and metabolism of these molecules, the common denominator can also be found in diseases or sudden changes in thyroid function and in liver damage, which should be analyzed in further observations. It is necessary to consider whether the relatively low concentration of cystatin C and high cholesterol in COVID-19 are part of the pathogenesis or a result of the disease. Nevertheless, cystatin C is probably much more significant than a marker of the glomerular filtration rate.

When analyzing different levels of NGAL in the studied groups, one should note that this protein is produced not only by the kidney but by many other cells and tissues, including neutrophils, lungs, stomach, colon, heart, or even adipocytes [22]. NGAL also performs differently concerning disease specificity—septic, nephrotoxic, or ischemic [23]. In oncogenesis, it can have both pro- and anti-tumor activity depending on the type of tumor [24]. The usefulness of NGAL in predicting the course of kidney disease is complicated as plasma and urinary NGAL respond to renal tubular damage; however, they differ in the size and structure of the molecule and provide different information in diagnosis and prognosis [25]. In our study, in the IG(−) group and in the C1 and C14 time-points in the COVID-19 group, a positive correlation between cystatin C and NGAL was observed. Serum or plasma NGAL and cystatin C are known to correlate with each other, as both molecules usually relate to kidney function; however, NGAL is strongly associated with the cells of innate immunity, and cystatin is related to the activity of most nucleated cells. Blood levels of NGAL are very susceptible to changes in the body caused by trigger factors, while the range of cystatin C values in both health and disease is much smaller and stretched over time. An ongoing infection and its consequences may significantly affect the concentration of NGAL, which is released mainly by neutrophils, and its levels should first be related to systemic inflammation. However, this does not exclude that it still retains some dependence on kidney function.

In most of the studies on COVID-19 patients so far, NGAL levels were evaluated mainly in urine, as the diagnostic significance of NGAL in this material has been repeatedly demonstrated in many other inflammatory and non-inflammatory diseases. It was shown that NGAL was elevated in the urine of COVID-19 patients at ICU admission who later developed AKI during their ICU stay, with the maximum urinary NGAL value in the first 48 h from admission. Moreover, the levels of this protein were correlated with the duration of mechanical ventilation [26]. Some other studies indicate that measurement of urinary NGAL, together with artificial intelligence (AI)-based chest computed tomography (CT) quantification, is worthy of application and may help clinicians to swiftly identify patients with a poor prognosis in COVID-19 [27,28]. Years before the appearance of SARS-CoV-2, the combined analysis of urinary and plasma NGAL levels in a mixed medical and surgical adult ICU in critically ill patients without pre-existing kidney disease or renal transplantation was performed to assess whether they could predict AKI occurrence up to 72 h post-ICU admission. The accuracy of NGAL both in urine and plasma appeared to improve as patients progressed through their ICU stay. The authors suggested that serial measurements of NGAL may be of added value in an ICU setting to predict AKI occurrence in critically ill patients [29,30,31]. Moreover, plasma NGAL levels evaluated at ICU admission predict acute kidney injury in adult patients [32], with additional accuracy when analyzed together with eGFR [33]. Taken together, plasma NGAL predictive values for AKI might refer only to patients in critical condition, independently of the primary disease, and should not be applied to patients suffering from mild to moderate symptoms. Importantly, recent evidence suggests that NGAL is not only a biomarker of heart and kidney diseases but modulates chronic inflammation and other processes that may lead to CKD progression [34].

As observed in our study, significantly elevated NGAL concentrations after asymptomatic infection might result from ongoing systemic inflammation or recent infection. The “patient zero” in Poland was recorded on 4 March 2020, and in Western Pomerania on 6 March 2020, so the asymptomatic infection must have occurred at this time. Blood was collected from hospital workers from mid-June 2020, and late antibodies, the immunoglobulin G class, appear two weeks to about one month after infection, depending on the type of infection, so it can be assumed that high levels of NGAL persist for at least one to three months after contact with the virus. The observed elevated mean concentration of plasma NGAL after asymptomatic infection (85.15 ng/mL), as well as during COVID-19 (78.94—104.54 ng/mL), is comparable to those observed in patients with chronic inflammation (98.19 ng/mL) when using the same material (plasma), method (ELISA), and supplier (R&D Diagnostics) [35]. The latest information is important as, indeed, the use of different materials (serum/plasma) and sets of different manufacturers can give different results. However, in various studies, one can observe a several-fold increase in the concentration of NGAL compared to healthy people or the effect of surgical and non-surgical treatment concerning an adequate reference group in certain inflammatory conditions.

NGAL present in the blood is released mainly by activated neutrophils, cells of the first line of the innate immunity, replaced with specialized adaptive lymphocytes and immunoglobulins in the next stages of the infection or reinfection. This reaction refers mainly to bacterial and fungal infections, but the role of neutrophils in responses to intracellular viruses has been less studied. Neutrophils are a major effector immune cell recruited to the lungs during respiratory viral infections, but their role is much more complex. Contrary to lymphocytes, which are lowered in ICU COVID-19 patients, the number of neutrophils, as well as the concentration of G-CSF (granulocyte colony-stimulating factor) in these patients, is significantly increased [36]. Elevated and activated neutrophils can cause immunopathology and exacerbate disease severity, especially in the lungs, as they are fundamental in the pathogenesis of many respiratory diseases [37]. Whether neutrophils have an active role in the antiviral immune response or bystander cells recruited to the lungs and airways by virus-induced inflammation has to be solved. NGAL might be crucial here, as, once released by neutrophils, it is associated with the occurrence and poorer outcome of renal and cardiac disease [37]. Moreover, since NGAL is released during the acute phase of infection, the long-lasting concentrations of this molecule after SARS-CoV-2 infection or COVID-19 remain an unsolved issue.

An increase in the concentration of plasma renalase in COVID-19 is important in explaining the biology of this still puzzling multi-functional molecule. The small size of the molecule (~36 kDa) suggests that it should be easily filtered by the glomeruli, especially in renal pathology, where this barrier is disrupted enough to enable the escape of large molecules, including albumin. As shown in CKD patients, serum renalase remains at a significantly higher level than in healthy persons, indicating that this protein is not only overproduced in CKD but is also stopped from escaping the blood by a yet unknown mechanism that occurs during kidney injury or dysfunction. This assumption is supported by the fact that both urinary renalase itself and after normalization to creatinine do not differ between healthy adults and renal patients [9,38]. In the present study, renalase levels were significantly higher in the COVID-19 patients from day 7 of observation until its end on day 28. There was no significant difference in renalase levels between the IG(−) control and IG(+) group or the C1 COVID-19 group. However, renalase levels in healthy people did not exceed an extremely low concentration of 0.59 ng/mL. A comparative discussion of renalase in COVID-19 patients is difficult because there is only one basic study on changes in renalase concentration in COVID-19 patients at the moment, which was released as a preprint. The study indicates that decreased plasma levels of renalase are associated with worse outcomes in COVID-19 [39]. The work is based on the assumption that renalase is a “survival factor”; hence, its deficiency results in worse prognosis and course of the disease. However, this claim may be somewhat exaggerated, as significantly elevated levels of this molecule are observed in people with kidney disease, and such significantly high concentrations may serve as a risk factor for major adverse cardiovascular events (MACE) and even death in renal patients [40].

As mentioned, we observed that renalase levels in the control group were almost undetectable. In individuals after asymptomatic infection, RNLS levels are increased, but not significantly, similar to COVID-19 patients immediately after admission to the hospital (C1 group). A significant increase in renalase was observed 7, 14, and 28 days after admission. In the first weeks of the disease, its severity and treatment are crucial for the course and prognosis; hence, changes in many factors, especially those related to the immune system, are obvious, but whether the plasma renalase increase results from overexpression and overproduction, increased release from erythrocytes, or “recycling” needs more investigation. Apart from exploring renalase’s role in cell signaling, this should be based on the analysis of renalase and NADPH-dependent cellular metabolism and changes in respiration cycles in which this molecule is involved.

There are currently many analyses of AKI occurrence in patients hospitalized due to COVID-19, especially in Wuhan and other parts of China, and other pandemic outbreaks: Italy, the United States, or Great Britain. However, no significant correlations are often observed regarding AKI in people hospitalized for COVID-19, even within hospitals in Wuhan [41]; the number of patients who develop symptoms of acute kidney injury ranges from less than 1% to as much as 90%. It seems that the severity of the disease may be crucial, but there are no standardized scales to assess it concerning many concomitant diseases. Most AKI incidences occur 14–22 days after admission to the hospital [41]. Monitoring kidney function in short-term hospitalized convalescents and people after asymptomatic infection is substantial to draw further conclusions, establish diagnostic and therapeutic standards, and then apply them in the future in accurate and targeted interventions. The roles of NGAL and cystatin C in predicting acute kidney injury during COVID-19 are already the subject of clinical trials, the results of which should be awaited. However, it is almost certain that some diagnostic regimens will have to be changed in the face of the pandemic and new consequences and general organ changes in patients after COVID-19 and asymptomatic infection. We have shown very similar observations for renalase. Again, it is necessary to focus on its contribution to the pathogenesis or course of kidney diseases as an immune and repair factor rather than a classical renal marker.

## 5. Conclusions

Kidney function assessed using creatinine and cystatin C levels over time is not affected by ongoing COVID-19 with mild to moderate severity. This function is also not related to the significantly increasing concentration of renalase during COVID-19. Moreover, both symptomatic and asymptomatic infection is associated with an increase in plasma NGAL concentration. Since NGAL still might be a biomarker and predictive factor in the development of inflammatory- and immune infiltration-related diseases, further observations on COVID-19 patients and people after asymptomatic infection are required to accurately determine the etiology, course, and effect of this phenomenon. It cannot be ruled out that asymptomatic infection causes some long-term changes similar to those observed in COVID-19. The current epidemic situation requires checking and adjusting many previously established methods and analytical parameters, and plasma NGAL alone should not be widely used to predict kidney damage, including AKI, or any other acute or chronic diseases.

### Limitations

It is not possible to accurately determine when people with IgG antibodies passed on the infection. Therefore, the results cannot be adjusted for the time that passed from the moment of infection to examination. It was also not possible to measure the bodyweight of the patients, including muscle mass and adipose tissue, as well as growth, which made it impossible to determine some derived parameters.

The study group was relatively small because, in many patients involved in the project, it was impossible to collect the material at the time-points strictly defined in the project and this study. MDRD was not calculated for each patient, taking creatinine as a benchmark and indicator of AKI. Moreover, we did not choose to diagnose AKI during this study; the blood was collected by separate qualified medical personnel and for research purposes only at the described time-points (0/1 day; 7 days; 14 days; 28 days) between 10 and 11 a.m. AKI was diagnosed as part of routine hospital diagnostic tests. We used the information about the occurrence or non-occurrence of AKI for statistical analysis, which showed no differences between the analyzed factors in people with or without AKI diagnosis.

The Bayesian network showed no significant influence of age, sex, diabetes, hypertension, or drugs on analyzed parameters, so they are not presented in the manuscript. These factors do not change over time for a given individual, and their influence was minimized in the COVID-19 group by using the tests for dependent variables. However, it is not easy to establish reference values, as this requires much greater group homogeneity. When analyzing independent groups (IG+, IG−, COVID-19), we cannot guarantee that these factors did not affect the results. Nonetheless, the statistical tools and methods used allowed us to obtain a reliable result.

## Figures and Tables

**Table 1:** Results of ELISA and biochemical measurements; data are shown as mean ± standard deviation and median (lower quartile–upper quartile); RNLS—renalase; NGAL—neutrophil gelatinase-associated lipocalin; CYS C—cystatin C; CREA—creatinine; UA—uric acid; GLU—glucose; TC—total cholesterol; HDL—high-density lipoprotein; TG—triglycerides; ALB—albumin.

GROUP/ PARAMETER	IG(−)	IG(+)	C1	C7	C14	C28
Sex (M/F)	18/10	9/19	16/14			
Age (years)	48 ± 9	42 ± 12	56 ± 15			
	49 (39–57)	42 (30–50)	58 (47–68)			
RNLS (ng/mL)	0.36 ± 0.09	4.62 ± 5.26	5.39 ± 4.63	9.66 ± 7.60	10.29 ± 7.39	8.51 ± 6.72
	0.39 (0.27–0.41)	2.86 (1.76–4.43)	3.55 (1.79–9.47)	7.22 (3.73–15.16)	8.82 (3.88–14.20)	6.20 (4.11–11.51)
NGAL (ng/mL)	25.84 ± 10.28	85.15 ± 36.28	78.94 ± 53.42	104.54 ± 57.14	89.11 ± 33.55	85.65 ± 33.83
	29.01 (17.29–33.75)	79.71 (58.56–102.81)	62.83 (50.66–84.26)	87.98 (69.96–122.58)	83.39 (64.42–107.84)	80.20 (58.34–113.88)
CYS C (mg/L)	0.87 ± 0.17	0.80 ± 0.23	0.71 ± 0.32	0.74 ± 0.31	0.70 ± 0.31	0.66 ± 0.26
	0.84 (0.75–0.96)	0.73 (0.66–0.87)	0.59 (0.50–0.90)	0.68 (0.50–0.84)	0.60 (0.49–0.87)	0.60 (0.45–0.86)
CREA (mg/dL)	0.76 ± 0.29	1.20 ± 0.96	1.20 ± 0.73	1.51 ± 1.16	1.24 ± 0.79	1.45 ± 1.02
	0.78 (0.65–0.91)	0.90 (0.53–1.83)	1.03 (0.75–1.63)	0.93 (0.66–2.3)	1.01 (0.70–1.91)	1.22 (0.70–1.91)
UA (mg/dL)	5.6 ± 0.8	5.5 ± 0.7	5.3 ± 1.1	5.7 ± 1.4	5.5 ± 0.9	5.5 ± 1
	5.5 (5.1–5.8)	5.5 (5.0–5.8)	5.1 (4.8–5.3)	5.3 (4.8–5.9)	5.3 (4.9–5.9)	5.2 (4.9–5.7)
GLU (mg/dL)	101 ± 19	82 ± 25	96 ± 31	110 ± 40	100 ± 32	90 ± 32
	93 (88–105)	78 (64–96)	88 (74–117)	116 (71–135)	99 (71–123)	81 (70–108)
TC (mg/dL)	192 ± 27	167 ± 17	211 ± 68	209 ± 65	229 ± 62	244 ±75
	192 (174–220	164 (157–182)	212 (151–265)	205 (164–241)	228 (180–275)	270 (178–299)
HDL (mg/dL)	73 ± 22	61 ± 10	70 ± 5	71 ± 6	73 ± 5	72 ± 7
	70 (56–85)	64 (58–68)	71 (68–74)	71 (69–75)	73 (71–75)	73 (70–77)
TG (mg/dL)	153 ± 29	97 ± 41	119 ± 46	174 ± 90	181 ± 99	149 ± 102
	148 (137–165)	87 (68–115)	111 (92–139)	144 (108–215	143 (113–230)	111 (81–180)
ALB (g/dL)	4.7 ± 1.2	4.8 ± 0.8	3.8 ± 0.5	3.8 ± 0.6	4.1 ± 0.8	4.2 ± 0.8
	4.2 (3.8–5.8)	5.0 (4.5–5.3)	3.8 (3.5–4.2)	3.7 (3.4–4.2)	4.3 (3.4–4.9)	4.4 (3.5–4.8)

## Supplementary Materials

The following supporting information can be downloaded at: https://www.mdpi.com/article/10.3390/diagnostics12010108/s1, Figure S1: Levels of biochemical parameters: glucose, triglycerides, total and HDL cholesterol, and albumin in the studied groups.

## References

[1] Bhattaryya S., Bramhachari P.V. (2020). Inflammation during Virus Infection: Swings and Roundabouts | SpringerLink. Dynamics of Immune Activation in Viral Diseases.

[2] Wan Y., Shang J., Graham R., Baric R.S., Li F. (2020). Receptor Recognition by the Novel Coronavirus from Wuhan: An Analysis Based on Decade-Long Structural Studies of SARS Coronavirus. J. Virol..

[3] Wiśniewska H., Skonieczna-Żydecka K., Parczewski M., Niścigorska-Olsen J., Karpińska E., Hornung M., Jurczyk K., Witak-Jędra M., Laurans Ł., Maciejewska K. (2021). Hepatotropic Properties of SARS-CoV-2—Preliminary Results of Cross-Sectional Observational Study from the First Wave COVID-19 Pandemic. J. Clin. Med..

[4] Taquet M., Geddes J.R., Husain M., Luciano S., Harrison P.J. (2021). 6-month neurological and psychiatric outcomes in 236379 survivors of COVID-19: A retrospective cohort study using electronic health records. Lancet Psychiatry.

[5] Kunutsor S.K., Laukkanen J.A. (2020). Renal complications in COVID-19: A systematic review and meta-analysis. Ann. Med..

[6] Ahmadian E., Khatibi S.M.H., Soofiyani S.R., Abediazar S., Shoja M.M., Ardalan M., Vahed S.Z. (2021). COVID-19 and kidney injury: Pathophysiology and molecular mechanisms. Rev. Med. Virol..

[7] Zhang N.-H., Cheng Y.-C., Luo R., Zhang C.-X., Ge S.-W., Xu G. (2021). Recovery of new-onset kidney disease in COVID-19 patients discharged from hospital. BMC Infect. Dis..

[8] Tissue Expression of RNLS-Summary-The Human Protein Atlas. https://www.proteinatlas.org/ENSG00000184719-RNLS/tissue.

[9] Wiśniewska M., Serwin N., Dziedziejko V., Marchelek-Myśliwiec M., Dołęgowska B., Domański L., Ciechanowski K., Safranow K., Pawlik A. (2019). Chronic kidney disease is associated with increased levels of renalase in serum and decreased in erythrocytes. Pol. Arch. Intern. Med..

[10] Passov A., Petäjä L., Pihlajoki M., Salminen U.-S., Suojaranta R., Vento A., Andersson S., Pettilä V., Schramko A., Pesonen E. (2019). The origin of plasma neutrophil gelatinase-associated lipocalin in cardiac surgery. BMC Nephrol..

[11] Cai L., Rubin J., Han W., Venge P., Xu S. (2010). The Origin of Multiple Molecular Forms in Urine of HNL/NGAL. Clin. J. Am. Soc. Nephrol..

[12] Gala-Bladzinska A., Kuzniewski M. (2013). Performance neutrophil gelatinase-associated lipocalin in clinical settings. Przegl Lek..

[13] Mishra J., Dent C., Tarabishi R., Mitsnefes M.M., Ma Q., Kelly C., Ruff S.M., Zahedi K., Shao M., Bean J. (2005). Neutrophil gelatinase-associated lipocalin (NGAL) as a biomarker for acute renal injury after cardiac surgery. Lancet.

[14] Bolignano D., Donato V., Coppolino G., Campo S., Buemi A., Lacquaniti A., Buemi M. (2008). Neutrophil Gelatinase–Associated Lipocalin (NGAL) as a Marker of Kidney Damage. Am. J. Kidney Dis..

[15] Hirsch R., Dent C., Pfriem H., Allen J., Beekman R.H., Ma Q., Dastrala S., Bennett M., Mitsnefes M., Devarajan P. (2007). NGAL is an early predictive biomarker of contrast-induced nephropathy in children. Pediatr. Nephrol..

[16] Testani J.M., Brisco M.A. (2016). Plasma NGAL: So, it Really Is Just Expensive Creatinine!. J. Am. Coll. Cardiol..

[17] Villa P., Jiménez M., Soriano M.-C., Manzanares J., Casasnovas P. (2005). Serum cystatin C concentration as a marker of acute renal dysfunction in critically ill patients. Crit. Care.

[18] Zhi H., Zhang M., Cui X., Li Y. (2019). Renal echography and cystatin C for prediction of acute kidney injury: Very different in pa-tients with cardiac failure or sepsis. Zhonghua Wei Zhong Bing Ji Jiu Yi Xue.

[19] Li Y., Yang S., Peng D., Zhu H.-M., Li B.-Y., Yang X., Sun X.-L., Zhang M. (2020). Predictive value of serum cystatin C for risk of mortality in severe and critically ill patients with COVID-19. World J. Clin. Cases.

[20] Klisic A., Kavaric N., Ninic A. (2020). Serum cystatin C levels are associated with triglycerides/high-density lipoprotein cholesterol ratio in adolescent girls ages between 16-19 years old. Eur. Rev. Med. Pharmacol. Sci..

[21] Qing X., Furong W., Yunxia L., Jian Z., Xuping W., Ling G. (2012). Cystatin C and asymptomatic coronary artery disease in patients with metabolic syndrome and normal glomerular filtration rate. Cardiovasc. Diabetol..

[22] Brisco M.A., Testani J.M. (2014). Novel renal biomarkers to assess cardiorenal syndrome. Curr. Heart Fail. Rep..

[23] Cernaro V., Bolignano D., Donato V., Lacquaniti A., Buemi A., Crascì E., Lucisano S., Buemi M. (2011). NGAL is a Precocious Marker of Therapeutic Response. Curr. Pharm. Des..

[24] Bolignano D., Donato V., Lacquaniti A., Fazio M.R., Bono C., Coppolino G., Buemi M. (2010). Neutrophil gelatinase-associated lipocalin (NGAL) in human neoplasias: A new protein enters the scene. Cancer Lett..

[25] Chakraborty S., Kaur S., Guha S., Batra S.K. (2012). The multifaceted roles of neutrophil gelatinase associated lipocalin (NGAL) in inflammation and cancer. Biochim. Biophys. Acta BBA Rev. Cancer.

[26] Komaru Y., Doi K., Nangaku M. (2020). Urinary Neutrophil Gelatinase-Associated Lipocalin in Critically Ill Patients with Coronavirus Disease 2019. Crit. Care Explor..

[27] He L., Zhang Q., Li Z., Shen L., Zhang J., Wang P., Wu S., Zhou T., Xu Q., Chen X. (2020). Incorporation of Urinary Neutrophil Gelatinase-Associated Lipocalin and Computed Tomography Quantification to Predict Acute Kidney Injury and In-Hospital Death in COVID-19 Patients. Kidney Dis..

[28] Harmon S.A., Sanford T.H., Xu S., Turkbey E.B., Roth H., Xu Z., Yang D., Myronenko A., Anderson V., Amalou A. (2020). Artificial intelligence for the detection of COVID-19 pneumonia on chest CT using multinational datasets. Nat. Commun..

[29] Matsa R., Ashley E., Sharma V., Walden A.P., Keating L. (2014). Plasma and urine neutrophil gelatinase-associated lipocalin in the diagnosis of new onset acute kidney injury in critically ill patients. Crit. Care.

[30] Mahmoodpoor A., Hamishehkar H., Fattahi V., Sanaie S., Arora P., Nader N.D. (2018). Urinary versus plasma neutrophil gelatinase-associated lipocalin (NGAL) as a predictor of mortality for acute kidney injury in intensive care unit patients. J. Clin. Anesthesia.

[31] Duda I., Krzych Ł. (2021). Plasma Neutrophil Gelatinase-Associated Lipocalin Is Useful for Predicting Mortality in Critically Ill Patients. J. Clin. Med..

[32] Koeze J., Van Der Horst I.C.C., Keus F., Wiersema R., Dieperink W., Kootstra-Ros J.E., Zijlstra J.G., Van Meurs M. (2020). Plasma neutrophil gelatinase-associated lipocalin at intensive care unit admission as a predictor of acute kidney injury progression. Clin. Kidney J..

[33] de Geus H.R.H., Bakker J., Lesaffre E.M.E.H., le Noble J.L.M.L. (2011). Neutrophil Gelatinase-associated Lipocalin at ICU Admission Predicts for Acute Kidney Injury in Adult Patients. Am. J. Respir. Crit. Care Med..

[34] Viau A., El Karoui K., Laouari D., Burtin M., Nguyen C., Mori K., Pillebout E., Berger T., Mak T.W., Knebelmann B. (2010). Lipocalin 2 is essential for chronic kidney disease progression in mice and humans. J. Clin. Investig..

[35] Otto G.P., Hurtado-Oliveros J., Chung H.-Y., Knoll K., Neumann T., Müller H.J., Herbsleb M., Kohl M., Busch M., Sossdorf M. (2015). Plasma Neutrophil Gelatinase-Associated Lipocalin Is Primarily Related to Inflammation during Sepsis: A Translational Approach. PLoS ONE.

[36] Velavan T.P., Meyer C.G. (2020). Mild versus severe COVID-19: Laboratory markers. Int. J. Infect. Dis. IJID Off. Publ. Int. Soc. Infect. Dis..

[37] Johansson C., Kirsebom F.C.M. (2021). Neutrophils in respiratory viral infections. Mucosal Immunol..

[38] Serwin N.M., Wiśniewska M., Cecerska-Heryć E., Safranow K., Skwirczynska E., Dołęgowska B. (2020). Serum-to-urine renalase ratio and renalase fractional excretion in healthy adults and chronic kidney disease patients. BMC Nephrol..

[39] Wang M., Guo X., Chun H.J., Lee A.I., Cha C., Gorelick F., Desir G.V. (2020). Decreased plasma levels of the survival factor renalase are associated with worse outcomes in COVID-19. medRxiv.

[40] Knop W., Serwin N.M., Cecerska-Heryć E., Grygorcewicz B., Dołęgowska B., Gomółka A., Wiśniewska M., Ciechanowski K. (2021). Elevated Levels of Renalase, the β-NAD(P)H Isomerase, Can Be Used as Risk Factors of Major Adverse Cardiovascular Events and All-Cause Death in Patients with Chronic Kidney Disease. Biomolecules.

[41] Zheng X., Zhao Y., Yang L. (2020). Acute Kidney Injury in COVID-19: The Chinese Experience. Semin. Nephrol..

